# From needles to pills: oral GLP-1 therapy enters the obesity arena

**DOI:** 10.1186/s40842-025-00245-5

**Published:** 2025-10-06

**Authors:** Gaetano Santulli

**Affiliations:** 1 Department of Medicine, Albert Einstein College of Medicine, New York City, NY 10461 USA; 2 International Translational Research and Medical Education (ITME) Consortium, Academic Research Unit, Department of Advanced Biomedical Sciences, “Federico II” University, 80131 Naples, Italy; 3 Department of Molecular Pharmacology, Wilf Family Cardiovascular Research Institute, Einstein Institute for Aging Research, Einstein-Mount Sinai Diabetes Research Center (ES-DRC), Einstein Institute for Neuroimmunology and Inflammation (INI), Fleischer Institute for Diabetes and Metabolism (FIDAM), Albert Einstein College of Medicine, New York City, NY 10461 USA

**Keywords:** Obesity, Oral GLP-1, Orforglipron, Semaglutide, Weight loss

## Abstract

Obesity remains a major global health challenge, driving type 2 diabetes, cardiovascular disease, and other complications. Despite effective lifestyle and surgical interventions, pharmacotherapy uptake has historically been limited. Injectable GLP-1 receptor agonists, including semaglutide and tirzepatide, have redefined expectations for medical obesity therapy, achieving 15–20% weight reductions in clinical trials. However, barriers such as injections, cost, and adherence limit their real-world use. In September 2025, two pivotal phase-3 trials of oral GLP-1 therapies were published. ATTAIN-1 evaluated orforglipron in 3,127 adults with obesity over 72 weeks, demonstrating a mean weight loss of 11.2%, ≥ 10% weight loss in 54.6%, and improvements in cardiometabolic parameters. OASIS-4 studied oral semaglutide 25 mg in 307 adults over 64 weeks, showing a mean weight loss of 13.6%, ≥ 10% weight loss in 63%, and favorable metabolic changes. Both agents exhibited gastrointestinal adverse events consistent with the GLP-1 class; additionally, orforglipron had five mild pancreatitis cases, while oral semaglutide reported mild dysesthesia. These results confirm that oral GLP-1 receptor agonists can produce clinically meaningful weight loss and metabolic benefits, expanding options beyond injectables. Real-world adoption will hinge on adherence, tolerability, long-term safety, patient preference, and payer coverage. Oral GLP-1 therapies represent a transformative step in obesity management, offering a convenient alternative that may broaden access and optimize individualized care.

For decades, the promise of a pill to treat obesity has hovered at the edges of imagination. This September, that vision came sharply into focus when *The New England Journal of Medicine* published two landmark phase-3 trials of oral GLP-1 receptor agonists [1, 2]. Orforglipron (ATTAIN-1^1^) and oral semaglutide 25 mg (OASIS-4^2^) both demonstrated double-digit weight loss, clinically meaningful cardiometabolic improvements, and safety profiles broadly consistent with injectable GLP-1 therapies [3–5].

The question now is not whether oral GLP-1s work, but how they will fit into the rapidly evolving therapeutic landscape for obesity.

## The obesity epidemic and the therapeutic gap

Obesity remains one of the most pressing global health crises of the 21st century. The World Health Organization estimates that over one billion people worldwide are living with obesity, and the prevalence continues to rise [6, 7]. Obesity is a major driver of type 2 diabetes, cardiovascular disease, liver disease, and certain cancers [8]. Yet, despite its scale and complexity, treatment options remain limited and often underutilized.

Lifestyle modification alone produces modest and often unsustained weight loss. Bariatric surgery is effective but invasive, resource-intensive, and not suitable for all patients [9–11]. Pharmacotherapy, once a peripheral player in obesity care, has surged into the spotlight with the arrival of highly effective injectable incretin-based therapies such as semaglutide and tirzepatide [3–5, 12, 13]. These agents have reshaped expectations of what medical therapy for obesity can achieve, with average weight reductions of 15–20% in clinical trials.

However, injections remain a barrier for some patients. Stigma, needle aversion, cost, and the logistics of storage and administration can limit uptake. The idea of a once-daily pill with comparable efficacy has therefore been a holy grail for the field. The new NEJM trials of oral orforglipron and high-dose oral semaglutide [1, 2] represent the closest we have come to that reality.

## The trials at a glance

### Orforglipron – The ATTAIN-1 trial

- Participants: 3,127 adults with obesity, without diabetes.
- Duration: 72 weeks.
- Mean BMI: ~37 kg/m².
- Results: Mean weight loss of − 11.2% at the 36 mg dose; 54.6% achieved ≥ 10% weight loss; -Improvements in waist circumference, blood pressure, and lipids.
- Safety: Gastrointestinal adverse events frequent but manageable; Five cases of mild pancreatitis; Modest increase in heart rate observed.

This was a large, multinational study with broad representation across sex, age, and ethnicity. Its scale enhances the generalizability of findings and provides a solid foundation for regulatory consideration [1].

### Oral semaglutide 25 mg – The OASIS-4 trial

- Participants: 307 adults with obesity, without diabetes.
- Duration: 64 weeks.
- Mean BMI: ~37 kg/m².
- Results: Mean weight loss of − 13.6%; 63% achieved ≥ 10% weight loss; Improvements in HbA1c, triglycerides, and inflammatory markers.
- Safety: Gastrointestinal side effects consistent with GLP-1 class; reports of mild dysesthesia in a small number of participants.

OASIS-4 was smaller and less diverse, with most participants being women and white. While the results are striking, the limited demographic scope tempers broad extrapolation [2].

### Shared findings and GLP-1 class effect

Both trials reaffirm several key points, including: Adults with obesity but without diabetes can achieve clinically meaningful weight reduction with oral GLP-1 therapy; the metabolic benefits extend beyond weight loss, with improvements in blood pressure, lipid profile, glycemic markers, and inflammatory parameters; the side-effect profile remains consistent with the GLP-1 class, dominated by gastrointestinal symptoms such as nausea, vomiting, and diarrhea [14, 15].

These results strengthen the evidence that the GLP-1 pathway is a central and powerful lever in obesity management [16–18]. Whether injectable or oral, the mechanism appears to deliver robust benefits that translate into improvements in cardiometabolic health.

### Key differences and their clinical relevance

Despite these commonalities, several differences between the two oral agents may shape their clinical adoption.

Trial Design and Population

- ATTAIN-1 was larger and geographically diverse, enhancing external validity [1].
- OASIS-4 was smaller, less diverse, and weighted toward female participants [2].

Administration

- Orforglipron is taken without special restrictions, a convenience advantage.
- Oral semaglutide requires ingestion in a fasting state with ≤ 120 ml of water, followed by a 30-minute wait before eating, drinking, or taking other medications — a regimen that may challenge adherence in real-world settings.

Lifestyle Interventions

- ATTAIN-1: participants received healthy lifestyle advice.
- OASIS-4: participants were prescribed a standardized 500 kcal daily deficit.

These differences may complicate cross-trial efficacy comparisons.

Safety Signals

- Orforglipron: pancreatitis (5 mild cases), heart-rate increase.
- Oral semaglutide: dysesthesia, an unusual finding that warrants further exploration.

These distinctions (see also Table 1) may appear subtle, but in clinical practice, they can significantly shape patient preference, adherence, and long-term outcomes.

**Table 1:** Head-to- head comparison: Orforglipron vs. Oral semaglutide (25 mg) vs. Injectable semaglutide (2.4 mg) vs. Tirzepatide (15 mg)

	Orforglipron (oral) ATTAIN-1	Oral Semaglutide 25 mg OASIS-4	Injectable Semaglutide 2.4 mg (*Wegovy*) STEP-1	Tirzepatide 15 mg (s.c.) SURMOUNT-1
Trial size (duration)	3,127 (72 weeks)	307 (64 weeks)	~ 1,961 (68 weeks)	~ 2,539 (72 weeks)
Population	Adults with obesity, no diabetes; mean BMI ~ 37; multinational, diverse	Adults with overweight/obesity, no diabetes; mean BMI ~ 37; mostly women, White	Adults with overweight/obesity, no diabetes; broad trial population	Adults with overweight/obesity, no diabetes; broad multinational enrollment
Co-intervention (lifestyle)	Lifestyle advice (not a standardized 500 kcal deficit)	Standardized 500 kcal/day deficit	Intensive lifestyle program (STEP)	Brief monthly lifestyle counselling
Mean % weight change (active arm)	−11.2% at 36 mg	−13.6%	−14.9%	−20.9%
Mean % weight change (placebo)	−0.9% to − 2.0%	−2.2%	−2.4%	−3.1%
Placebo-adjusted mean difference	−9.5 to − 11.5 percentage points	−11.4 percentage points	−12.4 percentage points	−17.8 percentage points
% achieving ≥ 10% weight loss	55–60%	63%	69.1%	~ 75%
% achieving ≥ 15% weight loss	~ 36–39%	~ 50%	50.5%	~ 57%
% achieving ≥ 20% weight loss	~ 18%	~ 30%	~ 33%	~ 50%+
GI adverse events	Frequent (nausea, vomiting, diarrhea)	Frequent, dose-related	Frequent, manageable with titration	Frequent, mild-to-moderate
Other safety signals	5 mild pancreatitis cases; modest HR increase	Mild dysesthesia; otherwise class-consistent	Class-consistent; ongoing monitoring for pancreatitis/thyroid	Class-consistent; long-term safety under study
Administration	Daily oral; no restrictions	Daily oral; fasting, ≤ 120 mL water, 30-min wait	Weekly subcutaneous injection; refrigeration, training	Weekly subcutaneous injection; titration, training
Discontinuations due to AEs	~ 10% (mostly GI)	Higher than placebo (mostly GI)	Modest; titration helps	6–7% at high dose
Approximate NNT for ≥ 10% WL	~ 3–4 (active ~ 55–60% vs. placebo ~ 17–20%)	~ 2 (63% vs. ~ 11%)	~ 2 (69.1% vs. 12%)	~ 2 (75% vs. ~ 13–15%)
Estimated annual cost (US 2025) & coverage	Not yet marketed; forecast ~$6,000–$9,000/year; coverage uncertain; may be restricted until long-term data mature	~$12,000–14,000/year; coverage limited; higher denial rates than injectables; Medicare exclusion persists	~$13,000–16,000/year; widely covered by commercial payers with PA; Medicare still excludes obesity drugs	~$13,000–16,000/year; coverage expanding; prior authorization common; insurers favor due to high efficacy
References	[1]	[2]	[3]	[4, 5]

AE, adverse event; BMI, body mass index; CI, confidence interval; GI, gastrointestinal; HR, heart rate; NNT, number needed to treat; NNH, number needed to harm; PA, prior authorization; s.c., subcutaneous; WL, weight loss

### Limits of cross-trial comparisons

It is tempting to place orforglipron and oral semaglutide head-to-head and crown a “winner.” Yet, the absence of direct comparison — particularly against injectable semaglutide (50 mg) [3] or tirzepatide [4, 5] (Table 1) — makes such judgments somehow premature. Different populations, designs, and co-interventions mean that any apparent differences in weight loss magnitude are suggestive but not definitive. For now, both agents should be viewed as part of a broader therapeutic class, with specific features that may suit different patient needs.

### Safety: beyond the GI story

Gastrointestinal side effects remain the defining safety signal for GLP-1 therapies. These are well recognized, generally dose-dependent, and often improve with time or titration adjustments [14, 15]. Yet, other signals merit close scrutiny, including pancreatitis, tachycardia, and dysesthesia. Though only five cases were reported with orforglipron and all were mild [1], any association with pancreatitis requires careful monitoring given the longstanding debate around GLP-1 biology and pancreatic risk. The modest increase in heart rate observed with orforglipron echoes findings from injectable GLP-1 trials. While the clinical significance is uncertain, patients with underlying arrhythmias or cardiovascular disease warrant particular caution. The sensory symptoms reported in the OASIS-4 trial [2] are atypical for this drug class and highlight the importance of broad surveillance for unexpected effects.

Hence, enthusiasm should not eclipse vigilance. Post-marketing surveillance and mechanistic research will be critical to ensuring that early safety signals are either contextualized or confirmed.

### Practical considerations in real-world use

Once these agents are available outside trial settings, several practical issues will determine their uptake and effectiveness:

- Adherence: Daily oral dosing may improve acceptance for patients reluctant to use injections, but administration restrictions (as with oral semaglutide) could be a barrier.
- Durability: Evidence from injectables suggests sustained benefit, but long-term data for oral formulations are still limited.
- Patient Preference: Some patients who have successfully used injectables may prefer to continue them rather than switch. Others may embrace the pill form eagerly.
- Cost and Coverage: Payer policies will play a decisive role. If priced similarly to injectables, the convenience of oral dosing may not offset high out-of-pocket costs.
- Clinical Integration: These agents must be embedded in a framework of multidisciplinary care — including dietary, behavioral, and, when appropriate, surgical strategies [19, 20].

### What comes next

Most likely, several unanswered questions will shape the trajectory of oral GLP-1 therapies, including: Can oral agents sustain weight loss and metabolic benefits over years, not just months? How do oral formulations compare directly against injectables semaglutide or tirzepatide in terms of efficacy, tolerability, and long-term outcomes? Will pancreatitis, heart rate increases, or other emergent signals prove clinically significant? Will pills meaningfully expand access for patients who currently forgo injectable therapy, or will cost remain the main bottleneck?

## Conclusion: cautious optimism with a mandate for rigor

The ATTAIN-1 and OASIS-4 trials mark a milestone in obesity medicine: proof that oral GLP-1 receptor agonists can achieve double-digit weight loss and meaningful cardiometabolic improvements in adults with obesity, without diabetes. These agents are not merely “me-too” versions of their injectable predecessors; they represent a potentially transformative option for patients who have long sought an effective oral therapy.

Yet, the path forward demands rigor. Oral GLP-1 molecules must prove durable benefits, clarify safety signals, and establish their place relative to powerful injectable agents. Adherence, real-world persistence, and payer coverage will ultimately determine their impact more than trial averages.

If these challenges are met, oral GLP-1s could become a cornerstone of obesity management — not replacing injectables or surgery, but broadening the therapeutic spectrum. For a field long constrained by stigma, limited options, and therapeutic inertia, that possibility is as exciting as it is overdue.
